# Effects of Heat-Treated Wood Particles on the Physico-Mechanical Properties and Extended Creep Behavior of Wood/Recycled-HDPE Composites Using the Time–Temperature Superposition Principle

**DOI:** 10.3390/ma10040365

**Published:** 2017-03-30

**Authors:** Teng-Chun Yang, Yi-Chi Chien, Tung-Lin Wu, Ke-Chang Hung, Jyh-Horng Wu

**Affiliations:** Department of Forestry, National Chung Hsing University, Taichung 402, Taiwan; tcyang.04@nchu.edu.tw (T.-C.Y.); moneyboy824@gmail.com (Y.-C.C.); tonywuwu22@gmail.com (T.-L.W.); d9833004@mail.nchu.edu.tw (K.-C.H.)

**Keywords:** heat treatment, wood/recycled-HDPE composite, physico-mechanical properties, creep behavior, time–temperature superposition principle

## Abstract

This study investigated the effectiveness of heat-treated wood particles for improving the physico-mechanical properties and creep performance of wood/recycled-HDPE composites. The results reveal that the composites with heat-treated wood particles had significantly decreased moisture content, water absorption, and thickness swelling, while no improvements of the flexural properties or the wood screw holding strength were observed, except for the internal bond strength. Additionally, creep tests were conducted at a series of elevated temperatures using the time–temperature superposition principle (TTSP), and the TTSP-predicted creep compliance curves fit well with the experimental data. The creep resistance values of composites with heat-treated wood particles were greater than those having untreated wood particles due to the hydrophobic character of the treated wood particles and improved interfacial compatibility between the wood particles and polymer matrix. At a reference temperature of 20 °C, the improvement of creep resistance (*ICR*) of composites with heat-treated wood particles reached approximately 30% over a 30-year period, and it increased significantly with increasing reference temperature.

## 1. Introduction

Wood-plastic composites (WPCs), in which lignocellulosic fillers act as a reinforcement, present many advantages, such as low cost, high specific strength and modulus, renewability, and biodegradability, compared with reinforced composites containing inorganic fillers (mineral fillers, glass fiber, etc.) [1]. However, the use of lignocellulosic fillers in polymer composites have several drawbacks as compared with inorganic fillers, including: (1) higher hygroscopicity, leading to dimensional changes due to their hydrophilic nature; (2) incompatibility between the hydrophilic lignocellulosic fillers and hydrophobic polymers; (3) non-uniform dispersion; and (4) lower thermal stability. Among these obstacles that limit the applications of WPCs in engineering materials, hygroscopicity, and incompatibility between the hydrophilic lignocellulosics and hydrophobic thermoplastics have been identified as major drawbacks. Therefore, to overcome these drawbacks, many studies have been conducted to modify the lignocellulosic materials by chemical approaches to decrease their hygroscopicity and improve their dimensional and thermal stabilities [2,3]. However, chemical agent modification has been the least convenient because it is chemically-based, time-consuming, fastidious to process, and not eco-effective. Therefore, one of the physical modifications, heat treatment, is a method that has been attractive to academic and industrial investigators to improve the hygroscopicity, dimensional stability, and biological resistance of lignocellulosic materials [4,5,6,7] without contaminating the environment. Some studies have indicated that the addition of heat-treated lignocellulosic fillers in WPCs could lead to an improvement of the water resistance and thermal stability of polymer composites [8,9,10,11]. Therefore, heat treatment could be a positive method to improve WPCs and, thereby, extend their use in wider engineering applications.

The main changes in the chemical composition of the lignocellulosics that occur during heat treatment are: (1) decomposition of hemicellulose and lignin; (2) carbohydrate depolymerization, which is accelerated by the formation of formic acid and acetic acid; and (3) increased lignin-carbohydrate cross-linking [12,13,14]. Furthermore, these changes after heat treatment create their own drawbacks, such as undesired color, reduction of mechanical properties, and unpleasant odors [15,16,17]. It is also important to investigate the temperature sensitivity of the WPC properties, such as its creep behavior, because WPCs exhibit a strong time–temperature dependent response. However, it is time-consuming and expensive to conduct full-scale creep tests at a normal time scale. In this study, an accelerated creep test based on the time–temperature superposition principle (TTSP) was implemented to predict the long-term creep response. Methods using this principle have been employed to confirm that TTSP is applicable to various WPCs [18,19,20,21,22].

To date, most studies have primarily focused on the physico-mechanical properties of polymer composites containing heat-treated lignocellulosic fillers, such as flexural properties, internal bond strength, morphology, and water resistance [12,15,16,23,24]. The polymers used as the polymer matrix in WPCs are often polyethylene (PE) and polypropylene (PP). The results from these studies indicated that reductions in water uptake and dimensional expansion, as well as increased biological durability, were observed when heat-treated lignocellulosic fillers were used in WPCs, while their mechanical properties were not significantly improved [12,24,25]. However, to the best of our knowledge, there is little available information on the creep behavior of heat-treated lignocellulosic filler/recycled-plastic composites. Additionally, few studies have been conclusively valid for creep properties of WPCs using TTSP. Therefore, in addition to detailed investigations related to their physico-mechanical properties, a main objective of the present study was to investigate the time–temperature dependent responses and TTSP-predicted creep behaviors of recycled-high density PE (rHDPE) filled with heat-treated wood particles. On the other hands, the TTSP-predicted curves were valid as compared with the experimental curves and discussed the suitability for the creep behavior of WPCs using TTSP.

## 2. Materials and Methods

### 2.1. Materials

Horng Gee Co., Ltd. (Changhua, Taiwan) kindly supplied the rHDPE (melting point: 130 °C; melt flow index: 4.2 g/10 min; density: 940 kg/m^3^). These plastic pellets were ground in an attrition mill to reduce their particle size to less than 20 mesh (850 μm) before the composite processing. Japanese cedar (*Cryptomeria japonica* D. Don) sapwood was sourced from the experimental forest of the National Taiwan University. Wood particles were prepared by hammer milling and sieving. Wood particles between 30 and 60 mesh (250–600 μm) were utilized in this investigation.

### 2.2. Heat Treatment of Wood Particles

The wood particles were placed in a conventional oven (CMF-25S, Fann Chern Industrial Co., Ltd., Taizhong, Taiwan) in a normal atmosphere, and heat treatments were conducted at 120, 160, or 200 °C for 2 h.

### 2.3. Processing of Composite Panels

Manufacturing WPCs: the flat-platen pressing process was applied, according to our previously described methods [3,26]. The weight ratio of oven-dried wood particles (moisture content <3%) to rHDPE powder was 50/50 (wt %). The expected density of the WPCs was 700 ± 50 kg/m^3^. The WPC samples had thickness values of 12 mm (for determining the physico-mechanical properties) and 3 mm (for determining the short-term and long-term creep behaviors). All of the WPCs were produced in a two-step pressing process as follows: (1) hot pressing (50 MPa) at 180 °C; and (2) finishing by cold pressing until the temperature of WPCs decreased to room temperature. In this study, untreated and heat-treated wood particles treated at 120, 160 and 200 °C were incorporated into rHDPE composites designated WPC_NT_, WPC_120_, WPC_160_ and WPC_200_, respectively.

### 2.4. Determining of the Composite Properties

Density, water absorption, thickness swelling, flexural properties, wood screw holding strength, and internal bond strength were determined according to the Chinese National Standard CNS 2215. Modulus of rupture (MOR) and modulus of elasticity (MOE) data have been obtained using the three-point static bending test with a loading speed of 10 mm/min and span of 180 mm (sample size: 230 mm × 50 mm × 12 mm). The wood screw holding strength and internal bond strength were determined on samples with dimensions of 50 mm × 50 mm × 12 mm at a tensile speeds of 1.5 and 2 mm/min, respectively. All samples were conditioned at 20 °C and 65% relative humidity for two weeks prior to testing, and at least five specimens of each blend were tested.

### 2.5. Evaluation of the Vertical Density Profile

The vertical density profiles of specimen (50 mm × 25 mm and 12 mm thickness) were measured at 0.04 mm intervals using a QTRS-01X X-ray density profiler (Quintek Measurement Systems, Knoxville, NT, USA). Eighteen specimens of each blend were tested.

### 2.6. Scanning Electron Microscopy

The morphologies of the wood particles and plastic in the composites were examined by scanning electron microscopy (SEM). Following an internal bond strength test, the fracture surfaces of the composites were dried and then imaged using a Hitachi TM-1000 (SEM, Hitachi, Tokyo, Japan) SEM instrument with an accelerating voltage of 15 kV. These samples were viewed perpendicularly to the fractured surface.

### 2.7. Short-Term Accelerated and Experimental Creep Tests

A dynamic mechanical analysis (DMA) (DMA 8000, PerkinElmer, Seer Green, UK) was conducted in the dual cantilever mode under isochronal conditions at frequencies of 4, 8, 12, or 16 Hz to determine the glass transition temperature (*T*_g_), and activation energy (*E*_a_) was calculated according to the TTSP methodology. The dimensions of these sample were 50 mm × 10 mm with a thickness of 3 mm. Tests were conducted in the range of −150 °C to −70 °C at a scanning rate of 1 °C/min. To predict the long-term creep behavior of the composites, the real-time short-term creep response at elevated temperatures, TTSP, was used. The creep compliance is following as:

*S*(*T*_ref_, *t*) = *S*(*T*_elev_, *t*/*α*_T_) (1)

where *S* is the creep compliance as a function of temperature and time, *T*_ref_ is the reference temperature, *T*_elev_ is the elevated temperature, and *α*_T_ is the shift factor. The activation energy of the glass transition relaxation and the master curve of creep compliance were also determined by DMA. Creep and creep recovery cycle tests were conducted at isotherms between 20 and 80 °C in intervals of 5 °C. A three-point bending mode with a span of 40 mm was used. For each isotherm, 20% of the average flexural strength was applied for 1 h, followed by a 1 h recovery period.

To validate the master curves derived from the short-term creep tests, a full-scale, experimental creep test was conducted. The applied stress was the same as that used in the short-term creep tests, and the mid-span strain values of samples with dimensions of 80 mm × 16 mm × 3 mm were recorded. The deflections were measured using a linear variable differential transducer (LVDT) mounted on an aluminum frame and placed at the mid-span of the samples. The samples were held at a temperature of 20 °C for a period of 90 days.

### 2.8. Theoretical Creep Models

Creep is the continuous accumulation of deflection over time when the material is subjected to a constant load. WPCs exhibit this type of viscoelastic behavior, and their creep strain mainly depends on the time, stress, and temperature. Therefore, the three main components of this strain, *ε*(*σ*, *T*, *t*), are presented as follows [27]:

*ε*(*σ*, *T*, *t*) = *ε*_e_(*σ*, *T*) + *ε*_ve_(*σ*, *T*, *t*) + *ε*_vp_(*σ*, *T*, *t*) (2)

where *ε*_e_(*σ*, *T*) is the reversible elastic deformation, *ε*_ve_(*σ*, *T*, *t*) is the reversible viscoelastic deformation, and *ε*_vp_(*σ*, *T*, *t*) is the irreversible viscoplastic deformation. Within the linear viscoelastic range of the materials, simple rheological models are appropriate. Among these models for WPCs, Burger’s model has often been used [18,28,29]. This model includes four elements that are combined with the Maxwell and Kelvin-Voigt models to quantitatively show the relationship between the morphology of the prepared materials and their creep behavior. The model is presented in the following equation:

(3) $$\varepsilon \left(t\right)=\frac{\sigma }{E_{M}}+\frac{\sigma }{E_{K}}\left[1-\exp\left(-\frac{E_{K}}{\eta_{K}}t\right)\right]+\frac{\sigma }{\eta_{M}}t$$

where *ε* is the strain at time *t*, *E*_M_, and *E*_K_ are the elastic moduli of the springs, *η*_M_ and *η*_K_ are the viscosities of the dashpots of the Maxwell and Kelvin-Voigt bodies, respectively, *σ* is the applied stress, and *t* is the elapsed time. *E*_M_ is associated with the instantaneous elastic strain that can be immediately recovered when the stress is removed. *E*_K_ and *η*_K_ determined the stiffness and viscous, or orientated, short term flow of the amorphous polymer chains. The ratio *η*_K_/*E*_K_ is designated as the retardation time, *τ*, which is a measure of the time required for the extension of the spring to its equilibrium length while being retarded by the dashpot. Finally, *η*_M_ contributes to the region of steady state creep, in which viscous flow is the predominant behavior [18]. An empirical mathematical model has often been applied to describe non-linear creep behavior. An example of this model is the Findley power law, and it was successfully used to fit the TTSP-predicted creep behavior for various WPCs [30,31]. The Findley power law has the form [32]:

*S*(*t*) = *S*_0_ + *at^b^* (4)

where *S*(*t*) is the time-dependent compliance, *S*_0_ is the instantaneous elastic compliance, *a* and *b* are constant numbers, and *t* is the elapsed time.

### 2.9. Analysis of Variance

All results were expressed in terms of the mean ± SD. The significance of the differences were calculated using Scheffe’s test; *p* < 0.05 was considered to be significant.

## 3. Results and Discussion

### 3.1. Density, Moisture Content, Water Absorption, and Thickness Swelling

**Figure 1:**
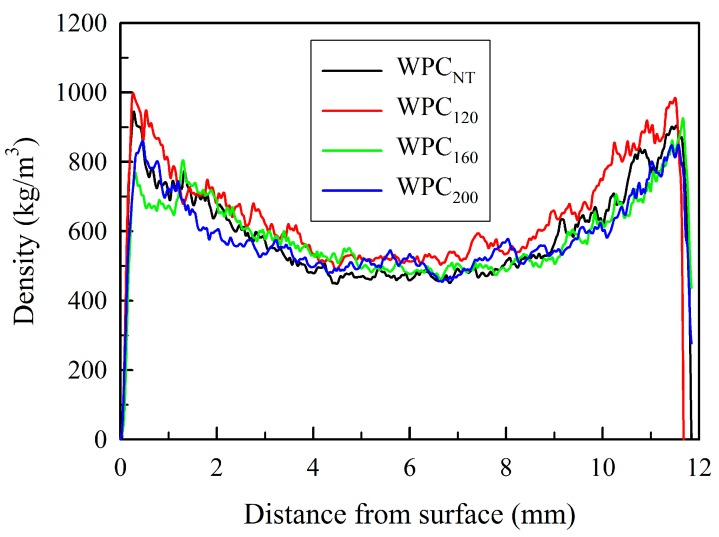
Vertical density profiles of WPCs with wood particles having different heat treatment temperatures.

Some of the physical properties of the unheated and heat-treated wood filled rHDPE composites are listed in Table 1. Generally, the density and moisture content values may directly impact the flexural properties of a polymer composite. The density of all of the samples ranged from 700 to 729 kg/m^3^, and there were no significant differences in the density values among all of the composites. As shown in Figure 1, the vertical density profiles of all of the composites resembles a flat “U-shape” that is similar to the typical vertical density profiles of particleboards produced by conventional hot pressing [33]. There were no significant differences in the vertical density profiles among all of the WPCs. However, the moisture content of the WPCs decreased with increasing heat treatment temperature. With heat-treated wood particles, the moisture content decreased significantly from 2.4% (WPC_NT_) to 2.1%, 1.8%, and 1.3% for WPC_120_, WPC_160_, and WPC_200_, respectively. It is well known that the moisture absorption of composites is mainly due to the gaps and flaws at the interfaces and the microcracks in the polymer matrix that are formed during processing [34], in addition to the hydrophilic nature of the wood particles. Furthermore, after 24 h of water immersion, the water absorption (WA), and thickness swelling (TS) values of the immersed samples were significantly influenced by the heat treatment level of the wood particles within the WPCs. The rHDPE composites with untreated wood particles (WPC_NT_) had the highest water absorption (10.0%) and thickness swelling (2.1%). The water absorption of composites has been attributed to the hydrogen bonding of water molecules to the free hydroxyl groups present in the cellulosic cell wall materials, especially hemicelluloses, and the diffusion of water into the wood particle-polymer matrix interface [35]. By contrast, the heat-treated wood/rHDPE composites showed lower values when compared with WPC_NT_. The WPC_160_ showed reductions in the WA and TS values of 24% and 19%, while WPC_200_ resulted in reductions of 30% and 28.6%, respectively. These results demonstrate that WPCs with heat-treated wood particles have higher water resistance and dimensional stability. These phenomena have been attributed to the modification of the wood particles in its structure and in its chemical composition, such as the removal of the hemicelluloses and the increase of the cellulose crystallinity [36,37]. Órfão et al. [38] reported that hemicelluloses start decomposing at 160 °C, through the hydrolysis of covalent bonds that link the hemicelluloses to lignin.

### 3.2. Flexural Properties, Wood Screw Holding Strength, and Internal Bond Strength

Table 2 presents the various mechanical properties of all of the heat-treated and untreated WPCs. The MOR, MOE, and wood screw holding strength of all heat-treated WPCs exhibited no significant differences when compared with WPC_NT_, while the internal bond strength was seriously affected by the heat treatment. The heat treatment of wood particles at 120–200 °C increased the internal bond strength values of the WPCs by 26.7%–33.3%, depending on the heat treatment temperature.

Comparison with WPC_NT_, however, showed that there were no significant changes of the MOR, MOE, and wood screw holding strength of WPCs with heat-treated wood particles. This finding could be related to interplay of two conditions. One is the formation of soluble acidic constituents (formic acid, acetic acid, etc.) from hemicellulose degradation [39]. These acids cause the depolymerization of the crystalline structure of the cellulose by breaking down the cellulose polymer chain into shorter chains. In addition, the separation of the lignin–hemicellulose copolymer and the depolymerization of the hemicellulose and amorphous cellulose were induced due to cleavage of the C–C and C–O linkages at the intra-polymer level [40]. Several investigators have reported that heat treatment reduces the MOE and MOR values of wood [41,42] and WPCs [24,43], especially treatment at high temperature because of depolymerization and shortening of the cellulose polymer chain. In addition, this phenomenon decreased the wood screw holding strength of the heat-treated WPCs that was attributed to the reduction of the ability of the wood particles to conform to the threads of the screw, which would otherwise transfer the load continuously along the threads [24].

**Figure 2:**
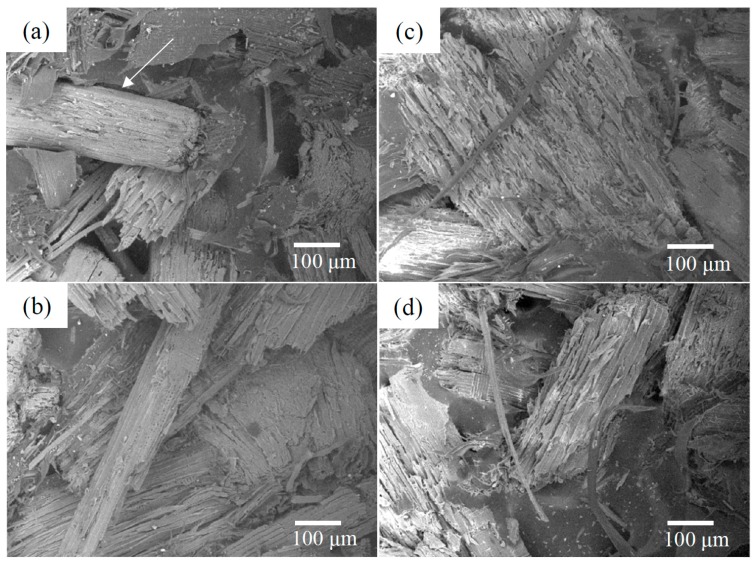
SEM micrographs of untreated and heat-treated WPCs. (**a**) WPC_NT_; (**b**) WPC_120_; (**c**) WPC_160_; and (**d**) WPC_200_. White arrow: gap between wood particle and polymer matrix.

The other condition is the compatibility of the wood particles, which is of great importance in achieving good adhesive interaction between the wood particles and the polymer matrix [44]. Figure 2 provides the SEM micrographs of the fracture surfaces of all of the WPCs. It can be seen that there is a remarkable difference in the wood particle/polymer matrix interactions of the WPC_NT_ and the heat-treated WPCs. No polymer matrix residue was left on the surface of the wood particles in the WPC_NT_. By contrast, in the all of the heat-treated WPCs, the wood particles were covered by layers of polymer matrix, in which the matrix material was being pulled out together with the particles. It can be safely concluded that all of the heat-treated WPCs failures occurred at the wood particle/polymer matrix interface as a result of a more effective compatibility between the wood particles and the polymer matrix. The heat-induced physical modifications resulted in hydrophobic wood particles, thus improving compatibility, which is a requirement for intermolecular hydrogen bonding and the wide polarity differences of the surfaces, increasing the wood/polymer bonding interactions [45]. This good bonding adhesion causes the two surfaces to stick together so that the stress can be transmitted between them, increasing the flexural properties (MOR and MOE) and wood screw holding strength values of WPCs with heat-treated wood particles. Taking these conditions into account, we hypothesize that the fact that there were no significant changes in the MOR, MOE, and wood screw holding strength values of the WPCs containing the heat-treated wood particles could be related to a counterbalance of the thermal decomposition of the wood reinforcement and the increased interfacial compatibility of the wood particles and polymer matrix.

The internal bond strength values of the WPCs significantly increased after heat treatment of the wood particles. The heat treatment of the wood particles improved the internal bond strength of the WPCs by 33.3% compared with the WPC_NT_. These results imply that the mechanical interlocking between the wood particles and polymer matrix was positively affected by the heat treatment of the wood particles. The lowest internal bond strength value of 1.5 MPa was found for WPC_NT_, while the heat-treated WPCs had the highest internal bond strength values of 2.0 MPa (Table 2). The improvement of the internal bond strength of the WPCs containing heat-treated wood particles is mainly attributed to the surface roughness of the wood particles and compatibility between the wood particles and polymer matrix. As shown in Figure 2, there were substantially more rough wood particles and fewer gaps between the wood particle and polymer matrix observed in WPCs with heat-treated wood particles. These results indicate that mechanical bonding of these two surfaces can occur due to physical interlocking. The rough surface increased the total interfacial area between the wood particles and polymer matrix, trapping the polymer in the cavities [44]. These mechanisms were responsible for the improved compatibility and mechanical bonding between the two surfaces, leading to the higher internal bond strength values of WPCs.

### 3.3. Extended Creep Behavior Using TTSP and Long-Term Creep Behavior

**Figure 3:**
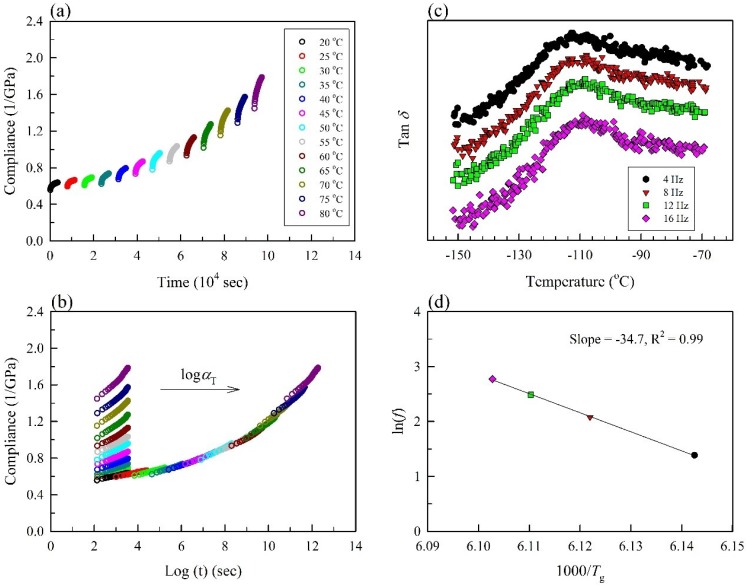
(**a**) The creep compliance of WPC_200_ at elevated temperatures in the actual test; (**b**) Unshifted and shifted creep compliance of WPC_200_ using a reference temperature of 20 °C against the test time in log scale; (**c**) Tan *δ* curves for a range of temperatures at a 1 °C/min; (**d**) Frequency in a log scale versus the inverse of *T*_g_.

To predict the long-term creep behavior of the composites, the short-term accelerated creep tests were conducted over a range of elevated temperatures using the TTSP method. Since WPC_200_ exhibited the lowest moisture content (ca. 1.3%) and an attractive dark color compared with WPC_NT_ that is preferred among Asian groups, this section of the paper has adopted WPC_200_ as an example to outline the shifted process of the creep compliance by the use of TTSP. The creep compliance at elevated temperatures in the actual time for the entire duration was shown in Figure 3a. The unshifted and shifted short-term creep compliance curves of WPC_200_ were plotted against the test time in a log scale at all of the temperatures tested (Figure 3b). The creep curves at elevated temperatures were shifted along the time axis to the right according to the reduced time using a shift factor (*α*_T_) calculated from the Arrhenius equation. The shift factor in a log scale, log *α*_T_, is related to the temperature and activation energy using the following equation:

(5) $${\log\alpha }_{T\;}=\;\frac{E_{a}}{R}\left(\frac{1}{T}-\frac{1}{T_{\mathrm{ref}}}\right)\times \text{log e}$$

where *α*_T_ is the horizontal shift factor, *E*_a_ is the activation energy of the glass transition relaxation (kJ/mol), *T* is the test temperature (K), *T*_ref_ is the reference temperature (K), and *R* is the universal gas constant (8.314 J/K·mol^−1^) [18,28]. *E*_a_ was estimated from the frequency dependence of the *T*_g_ of the rHPDE measured by DMA, therefore, the tested range of temperature must be overcome for the occurrence of molecular motion causing the transition [45]. As shown in Figure 3c, tan *δ* curves are plotted for a range of frequencies at a heating rate of 1 °C/min. From the peaks of the tan *δ* curves, the *T*_g_ values at different frequencies were determined. The *T*_g_ of WPC_200_ was −110.4 °C at a frequency of 4 Hz, and it increased with increases of the frequency. The *E*_a_ values can be calculated from the slope of the plot of ln(*f*) versus 1/*T*_g_ using the following equation:

(6) $$E_{a}\;=\;-R\frac{d\left(\ln\left(f\right)\right)}{d\left({1/T}_{g}\right)}$$

where *f* is frequency (Hz) and *T*_g_ is the glass transition temperature. The frequency on a log scale versus the inverse of *T*_g_ was plotted as shown in Figure 3d. A linear regression was performed on the slope, as validated by the coefficient of determination (*R*^2^) value that was greater than 0.99, and *E*_a_ was calculated to be 288.6 kJ/mol for WPC_200_, as shown in Table 3. According to this calculation, the *E*_a_ was higher in WPC_200_ (288.6 kJ/mol) compared with WPC_NT_ (280.2 kJ/mol), indicating that it requires more energy to mobilize the molecular chains of the rHDPE matrix during creep behavior.

**Figure 4:**
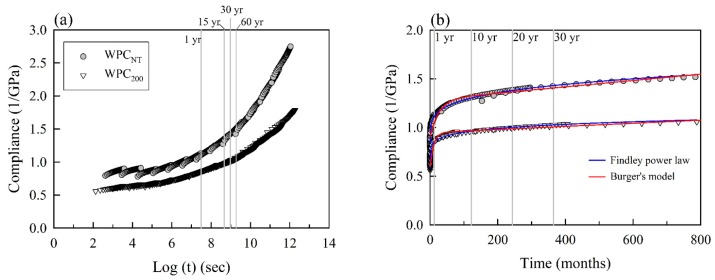
(**a**) The TTSP-predicted creep compliance values of WPC_NT_ and WPC_200_ in a log scale; (**b**) Creep master curves fitted with the Findley power law model and Burger’s model in a normal time.

The short-term accelerated compliance values of the various WPCs in a log time scale are shown in Figure 4a. It is noteworthy that the addition of the heat-treated wood particles remarkably reduced the compliance of WPC_200_ during the creep duration. This result reveals that the interface between the wood particles and the polymer matrix was improved for WPCs with heat-treated wood particles. Additionally, the creep compliance master curves of the various WPCs generated using shift factors were estimated from a constant activation energy assumption. Figure 4b shows the creep master curves in a normal time scale. There were two models used in this study to fit the creep curves for WPCs: Burger’s model and the Findley power law. Figure 4b shows that that both the Findley power law and Burger’s models fit the short-term accelerated creep compliance values of the WPCs over the elapsed time very well. Using WPC_NT_ as example, the *R*^2^ values of the Findley power law and Burger’s models were 0.994 and 0.960, respectively, although the power law model was clearly better. The fitted parameters of the Findley power law and Burger’s models are shown in Table 3. For the Burger’s model the instantaneous (*E*_M_) and retardant (*E*_K_) elastic modulus values of WPC_200_ were increased by approximately 50.9% and 8.7%, respectively, when compared with those of WPC_NT_, accounting for the enhanced adhesion of the polymer matrix/wood particles at the interface. The viscosity (*η*_K_) decreased by 56% for WPC_200_, and its retardation time (*τ*) decreased to 10.8 s compared with WPC_NT_ (26.7 s). These results reveal the increased polymer chain mobility of WPC_200_, indicating that the spring extends to equilibrium length at a shorter retardation time in the Kelvin-Voigt model. The other viscosity, *η*_M_, which is a measure of the long-term creep rate, increased from 3.07 × 10^3^ (WPC_NT_) to 6.30 × 10^3^ GPas in WPC_200_. The phenomenon indicates that WPC_200_ exhibited good creep resistance due to its lower creep rate compared with WPC_NT_. Additionally, for the Findley power law, the instantaneous elastic compliance (*S*_0_) and the parameter of viscous creep response (*b*) decreased to 0.51 GPa^−1^ and 0.12 in WPC_200_, respectively. The same results and explanation with Burger’s model indicate that the WPC with addition of the heat-treated wood particles exhibited the best creep resistance.

**Figure 5:**
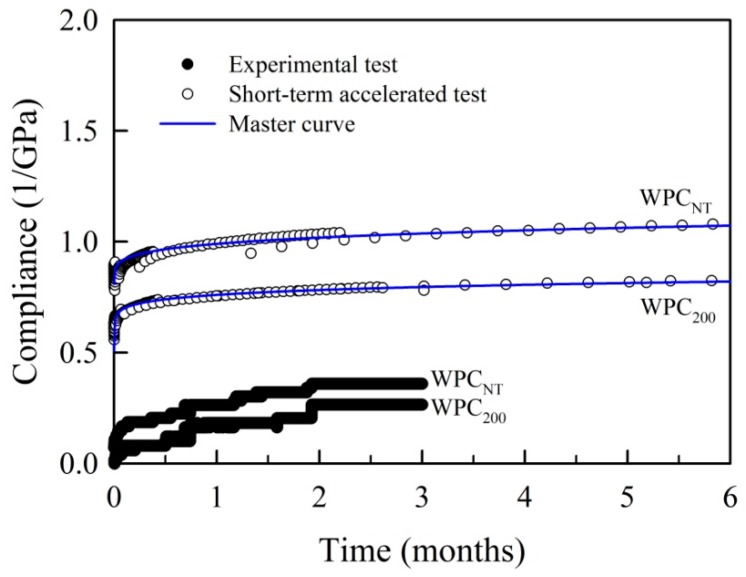
Comparison of the experimental creep compliance values and the master curves fitted with the Findley power law of WPC_NT_ and WPC_200_ in a normal time scale.

Due to the excellent fit with the TTSP-predicted compliance curves and simplified model, the Findley power law was selected to fit the master curves of all WPCs. Figure 5 presents the experimental and TTSP-predicted creep compliance curves of WPCs. This figure reveals that the creep compliance values of the experimental tests were lower than those of the master curves. There are many reasons that result in this difference, but two events are the most remarkable of all: one of the discrepancies is thermal expansion that occurs during the heating of the WPCs during the TTSP [46]; another is physical aging, which causes a stiffening of the WPCs with increasing time under the load in the long-term creep test [31,47]. Therefore, the short-term accelerated test of TTSP overestimated the degree of the creep compliance values, but its curves exhibited a trend that was consistent with the experimental test results.

To predict the lifetime creep behaviors of the WPCs, the instantaneous elastic compliance values (*S*_0_) and the predicted reductions in the modulus levels of all the WPCs at various reference temperatures over periods are tabulated in Table 4. The *S*_0_ and *b* parameters of WPC_NT_ declined with the increase of the reference temperature, while those of WPC_200_ showed no significant difference. The predicted compliance values at a reference temperature of 20 °C, WPC_NT_ were 1.11, 1.30, 1.38 and 1.43 GPa^−1^ at 1, 10, 20 and 30 years, respectively. The predicted WPC_200_ compliance values declined to 0.85, 0.95, 0.99 and 1.02 GPa^−1^ at 1, 10, 20 and 30 years, respectively. Both of the WPCs showed that their compliance values increased with increases of the reference temperature regardless of the elapsed time. Furthermore, to estimate the creep resistance of a sample under long-term conditions, the improvement of creep resistance (*ICR*) was calculated by the following equation [31]:

(7) $$ICR\;\left(\%\right)=\left[1-\frac{S{\left(t\right)}_{h}}{S{\left(t\right)}_{u}}\right]\times 100$$

where *S*(*t*)_h_ and *S*(*t*)_u_ are the time-dependent compliance values of the heat-treated and untreated WPCs at same reference temperature. As shown in Table 4, at a reference temperature of 20 °C, the *ICR* of WPC_200_ reached to 29% over a 30-year period, and it increased significantly with increasing reference temperature. According to the increased rate of the compliance (*b* value), the WPC_200_ showed a reduced *b* value (0.12) compared with WPC_NT_. Taken together, these results demonstrate that the creep resistance of the WPC was improved by the addition of heat-treated wood particles.

## 4. Conclusions

Compared with untreated wood particles, heat-treated wood particles exhibit better reinforcing effects on the water and creep resistance of recycled high-density polyethylene composites. The moisture content of WPCs decreased significantly with increasing heat treatment temperature, whereas the reductions in the water absorption and thickness swelling were observed for WPCs up to a heat treatment temperature of 160 °C. The MOR, MOE, and wood screw holding strength values of all of the WPCs with heat-treated wood particles exhibited no significant difference compared with untreated WPCs (WPC_NT_), but the internal bond strength values were greatly improved for the WPCs with wood particles that were heat-treated at temperatures of 120‒200 °C. The SEM micrographs of the fracture surfaces of WPCs showed that the surface of heat-treated wood particles became rough and were substantially covered by the polymer matrix. On the other hands, comparison of the experimental curve with short-term accelerated curve, TTSP-predicted creep compliance was overestimated but its trend was consistent with the experimental test result. Based on the predicted results, the WPCs with heat-treated wood particles exhibited improved creep resistance compared with WPC_NT_, indicating that the addition of heat-treated wood particles to WPCs significantly improved their dimensional stability and creep resistance due to the hydrophobic character of the treated wood particles and improved compatibility of the wood particles and polymer matrix at the interfacial surfaces. Accordingly, these composites could have an opportunity to replace conventional materials in building and construction applications.

## Figures and Tables

**Table 1:** Effects of heat treatment on density, moisture content, water absorption, and thickness swelling of WPCs.

WPCs	Heat Treatment Temperature (°C)	Density (kg/m^3^)	Moisture Content (%)	24 h Soaking	
				Water Absorption (%)	Thickness Swelling (%)
WPC_NT_	-	703 ± 13 ^A^	2.4 ± 0.2 ^C^	10.0 ± 2.1 ^B^	2.1 ± 0.1 ^B^
WPC_120_	120	704 ± 27 ^A^	2.1 ± 0.2 ^C^	8.7 ± 1.9 ^B^	1.9 ± 0.4 ^B^
WPC_160_	160	729 ± 22 ^A^	1.8 ± 0.2 ^B^	7.6 ± 1.7 ^A^	1.7 ± 0.4 ^A^
WPC_200_	200	700 ± 30 ^A^	1.3 ± 0.3 ^A^	7.0 ± 1.5 ^A^	1.5 ± 0.2 ^A^

Values are mean ± SD (*n* = 5). Different superscript letters within a column indicate significant differences (*p* < 0.05).

**Table 2:** Effects of heat treatment on flexural properties, wood screw holding strength, and internal bond strength of WPCs.

WPCs	Flexural Properties		Wood Screw Holding Strength (N)	Internal Bond Strength (MPa)
	MOR (MPa)	MOE (GPa)		
WPC_NT_	16.7 ± 1.9 ^A^	1.7 ± 0.2 ^A^	788 ± 62 ^A^	1.5 ± 0.3 ^B^
WPC_120_	17.8 ± 1.5 ^A^	1.8 ± 0.2 ^A^	705 ± 53 ^A^	2.0 ± 0.2 ^A^
WPC_160_	17.1 ± 0.9 ^A^	1.7 ± 0.1 ^A^	785 ± 50 ^A^	2.0 ± 0.3 ^A^
WPC_200_	15.8 ± 1.1 ^A^	1.6 ± 0.1 ^A^	755 ± 63 ^A^	1.9 ± 0.1 ^A^

Values are mean ± SD (*n* = 5). Different superscript letters within a column indicate significant differences (*p* < 0.05).

**Table 3:** Parameters of Burger’s model and Findley power law model for TTSP-predict creep compliance values of WPC_NT_ and WPC_200_ at a reference temperature of 20 °C.

Model	Parameters	WPC_NT_	WPC_200_
Burger’s model	*E*_M_ (GPa)	1.10	1.66
	*E*_K_ (GPa)	2.65	2.88
	*η*_K_ (GPas)	70.66	31.11
	*η*_M_ (GPas)	3.07 × 10^3^	6.30 × 10^3^
	*τ* (s)	26.7	10.8
	*R*^2^	0.974	0.960
Findley power law model	*S*_0_ (GPa^−1^)	0.81	0.51
	*a*	0.18	0.25
	*b*	0.21	0.12
	*R*^2^	0.987	0.994
Activation energy (kJ/mol)		280.2	288.6

**Table 4:** The predicted reductions in modulus of WPC_NT_ and WPC_200_ at various reference temperatures.

Code	Reference Temperature (°C)	*S*_0_ (GPa^−1^)	*a*	*b*	*R*^2^	*S*(*t*) (GPa^−1^)				*ICR* (%)			
						Time (Years)				Time (Years)			
						1	10	20	30	1	10	20	30
WPC_NT_	20	0.81	0.18	0.21	0.987	1.11	1.30	1.38	1.43	-	-	-	-
	25	0.79	0.30	0.18	0.992	1.26	1.50	1.59	1.66	-	-	-	-
	30	0.77	0.44	0.17	0.996	1.44	1.76	1.89	1.97	-	-	-	-
	35	0.75	0.62	0.16	0.998	1.67	2.08	2.24	2.34	-	-	-	-
	40	0.74	0.85	0.16	0.998	2.00	2.57	2.78	2.92	-	-	-	-
WPC_200_	20	0.51	0.25	0.12	0.994	0.85	0.95	0.99	1.02	24	27	28	29
	25	0.50	0.33	0.12	0.996	0.94	1.09	1.14	1.17	25	28	29	29
	30	0.51	0.40	0.12	0.998	1.05	1.22	1.28	1.32	27	31	32	33
	35	0.50	0.51	0.12	0.998	1.19	1.41	1.48	1.53	29	33	34	34
	40	0.50	0.64	0.12	0.999	1.36	1.64	1.74	1.80	32	36	38	38

*S*(*t*) = *S*_0_ + *at^b^*, where *S*(*t*) is the time-dependent compliance value, *S*_0_ is the instantaneous elastic compliance value, and *a* and *b* are constant numbers.
